# Opportunistic detection of atrial fibrillation in subjects aged 65 years or older in primare care: a randomised clinical trial of efficacy. DOFA-AP study protocol

**DOI:** 10.1186/1471-2296-13-106

**Published:** 2012-10-30

**Authors:** LuisÁ Pérula-de-Torres, MiguelÁ Martínez-Adell, Virginia González-Blanco, José M Baena-Díez, Enrique Martín-Rioboó, Juan M Parras-Rejano, Jesús González-Lama, Remedios Martín-Alvarez, Roger Ruiz-Moral, JoséÁ Fernández-García, Modesto Pérez-Díaz, Joaquin Ruiz-de-Castroviejo, Carlos Pérula-de-Torres, Antonio Valero-Martín, Ana Roldán-Villalobos, Margarita Criado-Larumbe, Emili Burdoy-Joaquín, Montserrat Coma-Solé, Mercè Cervera-León, Lluís Cuixart-Costa

**Affiliations:** 1 Unidad Docente de Medicina Familiar y Comunitaria de Córdoba, Instituto Maimónides de Investigación Biomédica de Córdoba (IMIBIC)/Hospital Universitario Reina Sofía/Universidad de Córdoba, Avda. Menéndez Pidal, s/n, Córdoba, 14004, Spain; 2 Centro de Salud Argentona, Consorci Sanitari del Maresme, C/Joan Fuster I Ortells, 1, Argentona, Barcelona, 08310, Spain; 3 Unidad Docente de Medicina Familiar y Comunitaria de Córdoba, Servicio Andaluz de Salud, C/Dr. Blanco Soler, s/n, Córdoba, 14004, Spain; 4 Centro de Salud La Marina. SAP Esquerra de Barcelona, Institut Català de la Salut, C/ Amnistia Internacional, 9, Barcelona, 08038, Spain; 5 Centro de Salud Fuensanta, Instituto Maimónides de Investigación Biomédica de Córdoba (IMIBIC)/Hospital Universitario Reina Sofía/Universidad de Córdoba, Avda. Menéndez Pidal, s/n., Córdoba, 14004, Spain; 6 Centro de Salud Peñarroya-Pueblonuevo, Instituto Maimónides de Investigación Biomédica de Córdoba (IMIBIC)/Hospital Universitario Reina Sofía/Universidad de Córdoba, Avda. Menéndez Pidal, s/n., Córdoba, 14004, Spain; 7 Distrito Sanitario Córdoba Sur, Instituto Maimónides de Investigación Biomédica de Córdoba (IMIBIC)/Hospital Universitario Reina Sofía/Universidad de Córdoba, Complejo Municipal Los Santos. Carretera Córdoba-Málaga s/n., Lucena, Córdoba, 14900, Spain; 8 CAP Vallcarca, CatSalut, Av. Hospital Militar, 169-205, Barcelona, 08023, Spain; 9 Unidad Docente de Medicina Familiar y Comunitaria de Córdoba, Instituto Maimónides de Investigación Biomédica de Córdoba (IMIBIC)/Hospital Universitario Reina Sofía/Universidad de Córdoba, Avda. Menéndez Pidal, s/n., Córdoba, 14004, Spain; 10 Centro de Salud Villarrubia-Azahara, Instituto Maimónides de Investigación Biomédica de Córdoba (IMIBIC)/Hospital Universitario Reina Sofía/Universidad de Córdoba, Avda. Menéndez Pidal, s/n., Córdoba, 14004, Spain; 11 Distrito Sanitario Córdoba-centro, Servicio Andaluz de Salud, Calle Dr. Blanco Soler, 4, Córdoba, 14004, Spain; 12 Hospital Regional Universitario Reina Sofía, Servicio Andaluz de Salud, Avda. Menéndez Pidal s/n, Córdoba, 14004, Spain; 13 Centro de Salud Villavisiosa de Córdoba (Córdoba), Instituto Maimónides de Investigación Biomédica de Córdoba (IMIBIC)/Hospital Universitario Reina Sofía/Universidad de Córdoba, Avda. Menéndez Pidal, s/n, Córdoba, 14004, Spain; 14 Centro Salud Villafranca (Córdoba), Instituto Maimónides de Investigación Biomédica de Córdoba (IMIBIC)/Hospital Universitario Reina Sofía/Universidad de Córdoba, Avda. Menéndez Pidal, s/n, Córdoba, 14004, Spain; 15 Centro de Salud de Huerta de la Reina (Córdoba), Instituto Maimónides de Investigación Biomédica de Córdoba (IMIBIC)/Hospital Universitario Reina Sofía/Universidad de Córdoba, Avda. Menéndez Pidal, s/n, Córdoba, 14004, Spain; 16 Unidad Docente de Medicina Familiar y Comunitaria de Córdoba, Instituto Maimónides de Investigación Biomédica de Córdoba (IMIBIC)/Hospital Universitario Reina Sofía/Universidad de Córdoba, Av. Menéndez Pidal, s/n, Córdoba, 14004, Spain; 17 Centro de Salud Collblanch, Consorci Sanitari Intergral, C/Creu Roja, 18, L′Hospitalet de Llobregat, Barcelona, 08904, Spain; 18 Centro de Salud Les Planes, Institut Català de la Salut, Av. Barcelona, 62, Sant Joan Despí, Barcelona, 08970, Spain; 19 Centro de Salud Roger de Flor, Equipo de Atención Primaria Dreta de l′Eixample, C/Roger de Flor, 194, Barcelona, 08013, Spain

**Keywords:** Atrial fibrillation, Screening, Opportunistic case finding, Secondary prevention, Primary care

## Abstract

**Background:**

Clinical Practice Guidelines recommend using peripheral blood pulse measuring as a screening test for Atrial Fibrillation. However, there is no adequate evidence supporting the efficacy of such procedure in primary care clinical practice. This paper describes a study protocol designed to verify whether early opportunistic screening for Atrial Fibrillation by measuring blood pulse is more effective than regular practice in subjects aged 65 years attending primary care centers.

**Methods/design:**

An cluster-randomized controlled trial conducted in Primary Care Centers of the Spanish National Health Service. A total of 269 physicians and nurses will be allocated to one of the two arms of the trial by stratified randomization with a 3:2 ratio (three practitioners will be assigned to the Control Group for every two practitioners assigned to the Experimental Group). As many as 12 870 patients aged 65 years or older and meeting eligibility criteria will be recruited (8 580 will be allocated to the Experimental Group and 4 290 to the Control Group). Randomization and allocation to trial groups will be carried out by a central computer system. The Experimental Group practitioners will conduct an opportunistic case finding for patients with Atrial Fibrillation, while the Control Group practitioners will follow the regular guidelines. The first step will be finding new Atrial Fibrillation cases. A descriptive inferential analysis will be performed (bivariate and multivariate by multilevel logistic regression analysis).

**Discussion:**

If our hypothesis is confirmed, we expect Primary Care professionals to take a more proactive approach and adopt a new protocol when a patient meeting the established screening criteria is identified. Finally, we expect this measure to be incorporated into Clinical Practice Guidelines.

**Trial registration:**

The study is registered as NCT01291953 (ClinicalTrials.gob)

## Background

Atrial fibrillation (AF) is defined as a cardiac arrhythmia involving irregular atrial activation, uncoordinated atrial systole and ineffective ventricular filling. ECG shows no P wave but displays fast fibrillation waves different in form, size and rhythm that lead to an irregular ventricular response. Echocardiogram shows no mitral A wave valvular movement [1]. AF is the most frequent arrhythmia in clinical practice and a growing challenge to public health due to population aging. The estimated prevalence of AF is 0.4-1% in the general population and increases with age up to 5-6% in the population aged > 65 years, and 8% in subjects aged 80 years or older [2,3]. Similarly, while the incidence of AF is < 0.1% per year in subjects aged < 40 years, it increases to above 1.5% per year in women and 2% in men aged > 80 years [4]. The incidence of AF was expected to increase 5-fold by the year 2010 [5-8]. According to the Framingham Heart Study, lifetime risk for AF is 1 in 4 in men and women aged 40 years or older. Lifetime risks remain greater even in absence of congestive heart failure or myocardial infarction (1 in 6) [9]. AF is one of the fastest-growing cardiovascular epidemics of the 21^st^ century, as it is one the major causes of morbidity and mortality, and it increases death risk, congestive heart failure and the risk for embolisms –including strokes [6,10-12]. One in every six strokes occur in subjects with AF [13,14], and the increased risk for ictus depends on the number of additional risk factors. In addition, the quality of life of these patients is severely impaired, mainly due to their inability to perform daily activities as a result of the risk of exacerbation of symptoms [15]. Over the last 20 years, admissions for AF have increased by 66% as a result of population aging, the increasing prevalence of chronic heart diseases, the more frequent diagnosis by ambulatory control mechanisms, and other factors [16].

A third of patients have asymptomatic AF. AF evolves from rare asymptomatic episodes to longer and more frequent strokes until it becomes permanent. Occasionally, AF is detected after an ECG performed during a hospitalization without a principal discharge diagnosis of AF [1]. The treatment of these patients represents a challenge continuously revised by experts [17]. The risk for cardioembolic processes in these patients significantly decreases with preventive treatment with oral anticoagulant drugs, which reduce relative risk by 68% and relative mortality by 2.5%, as compared with placebo [1].

Before recommending a screening test, a set of epidemiologic requirements should be met. The goal is to achieve a significant improvement in the prognosis of AF, since the decrease in the associated morbidity and mortality rates is greater when screening is performed. Early screening for AF might be highly beneficial for patients. The systematical population screening –especially in the elderly, as they are at a greater risk for AF and its complications–would be of great interest. The AF AWARE (Atrial Fibrillation Awareness And Risk Education) study, performed in 2009 in 11 countries with 810 cardiologists and 825 patients with AF concluded that early AF screening and treatment may help reduce severe AF-related risks [18]. Some studies have assessed the role of screening for AF, although it is more frequently detected and diagnosed when it becomes symptomatic [19-24]. Fitzmaurice et al [25] studied the cost-effectiveness of systematic screening versus the performance of ECG, and they concluded that blood pulse measuring is the best practice in cost-effectiveness terms; finally, they suggested the need for further studies assessing the efficacy of early screening for AF versus regular clinical practice. The practice of screening for AF in the elderly meets most of the criteria established by Wilson & Jungner to evaluate its implementation [26]: it is a usual and comprehensive process; it can be detected by simple procedures (blood pulse measuring is inexpensive, simple and harmless); diagnosis of AF can be confirmed by a simple and highly sensitive and specific test (ECC); screening for AF is free of iatrogenic effects, and the risk for complications or side effects as stroke can be significantly reduced with the available treatments. Two screening procedures have been proposed: opportunistic screening (performing screening when the practitioner sees a patient for any reason), or systematic (encouraging the patient to undergo a screening). There is evidence that the opportunistic option is the most appropriate [27].

The most recent Clinical Practice Guidelines recommend measuring blood pulse to test for suspected AF, and performing ECG to confirm a diagnosis of AF [28]. However, there is not adequate evidence supporting this recommendation, especially in the context of Primary Care.

### Research objectives

The three main aims of this study are:

To establish the percentage of patients with AF identified “de novo” by radial pulse assessment as compared to those discovered by regular clinical practice.

To know the amount of asymptomatic AF patients, and the symptoms and clinical signs showed by symptomatic AF patients.

To identify the different AF typologies following expert panels’ classifications.

Complementary objectives are:

To know the socio-demographics of the population with AF.

To identify health problems associated with this condition (comorbidity and risk factors).

To know the previous treatment followed by these patients.

## Methods/design

### Design of the study

**Figure 1:**
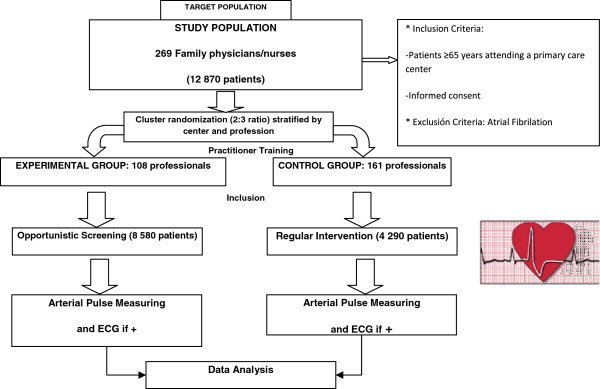
Design of DOFA-AP study.

A open, cluster, multicenter, two-arm, individual randomization, controlled trial of the efficacy of performing opportunistic screening versus regular clinical practice (Figure 1).

### Setting

Spanish National Health system. Primary Care physicians and nurses working in health centers in Barcelona, Córdoba and Badajoz. The study will have a duration of 12 months; fieldwork will have a duration of 5 months.

### Participants

The selection criteria were:

* Eligibility Criteria:

Men and women aged ≥ 65 years attending their health center for any reason.

* Exclusion Criteria:

Previous AF diagnosis (prevalent cases): we will measure the characteristics of patients previously diagnosed with AF and process the data obtained as existing cases, for statistic purposes.

### Sample size

The primary endpoint will be the percentage of AF cases; sample size estimation was performed according to the results obtained by Fitzmaurice DA [25] in its trial of effectiveness, using EPIDAT 3.1 software [29] and within the following criteria:

Risk in exposed patients: 1.63%; risk in non-exposed: 1.012%, expected relative risk: 1.62%.

Controls –CG– per case –EG–: 0.5 (1:2 ratio). With this ratio, we intend to optimize and make the study feasible, as it will be more difficult to identify and recruit controls.

Confidence level: 95.0%; accuracy: 80%; the resulting sample size is 7 722 subjects for the EG, and 3 861 subjects for the CG. As we estimated a non-response rate of 10%, we adjusted the formula Nf= Ni [1/(1-R)]=7 722 [1/(1-0,10)]= 8 580 (GE) and del GC= Ni [1/(1-R)]=3 861 [1/(1-0,10)]= 4 290. Consequently, the total N= 8 580 + 4 290=12 870 subjects. As this is a cluster design study and according to the study referenced above [25], at least 100 health professionals should recruit at least 12 000 patients. Since the probability of finding patients meeting the eligibility criteria is much lower for the CG than for the EG, stratified randomization will be used to assign at least 200 primary care health professionals to the two groups, with a 2:3 ratio. In other words, there will be three CG health practitioners for every two EG practitioners.

Patient selection will be performed by consecutive sampling.

### Randomisation

Researchers will be assigned to one of the study groups by stratified randomization based on the center and the type of health professional (GP/nurse) using EPIDAT 3.1 software [29].

### Interventions and comparative analysis

**-** EG: All patients seen by the health professionals involved in the present study will undergo active screening for AF, regardless of the reason of their visit. Therefore, the health professional will adopt a “proactive” attitude of opportunistic case finding, either because the professional identifies “silent” AF cases, or because screening for AF was performed, while the patient presented AF-associated symptoms, signs or pathologies.

-CG: Any patient presenting AF-accompanying symptoms (dyspnea, chest pain, palpitations or dizziness) or AF-induced sequels (stroke, transient ischemic attacks, peripheral embolism, heart failure or other associated health disorders) will be eligible. Once the practitioner identifies any of these symptoms, they will perform a screening for AF by measuring the patient’s blood pulse.

### Measurement Instruments and action program

1. A Data Collection Form (DCF) and a Procedure Manual including a Clinical Protocol based on an updated Practice Guideline will be designed [30].

2. A pilot study has already been conducted on a sample of 20 randomized patients in five health centers located in Barcelona and Córdoba, where five GP and five nurses participated. The target population was willing to undergo the test. The data collected by the DCF allowed practitioners to identify two cases of undiagnosed asymptomatic AF.

3. Communication Plan and Recruitment of Health Professionals: the project managers of the study were in charge of the dissemination of the study; in addition, a presentation of the study was performed in different health centers in Barcelona, Córdoba and Badajoz. A total of 269 health professionals at 28 health centers agreed to participate in the study (162 GPs and 107 nurses).

4. Training Plan. A presentation will be given to familiarize the participating practitioners with the study protocol. All participants will take an on-line course on how to approach patients with AF (http://www.semfyc.es/pfw_files/cma/Agenda/Sesiones_multimedia/Documentos/Diptico_referentes_fa.pdf). On the first stage, we intend to designate a practitioner as the expert in FA. The practitioner selected will take an online course to acquire the abilities and skills necessary to be able to give a training session to his/her partners.

### Measurements and Variables

The variables measured are shown in Table 1.

**Table 1:** Variables of the Study

**Variables**	**Definition**	**Measure scale**
**Independet**		
-Study Groups	EG: Opportunistic detection of AF	Dichotomous Qualitative
	CG: regular approach	
-Practitioner	Physician vs. Nurse	Dichotomous Qualitative
**-Sociodemographic:**		
Age	≥ 65 years	Quantitative Discrete
Sex	Man/woman	Dichotomous Qualitative
Marital Status	bachelor, married, widow/er, divorced	Qualitative Polytomous
Residence	Urban (≥ 10.000 population)/Rural (<10.000 population)	Dichotomous Qualitative
**-Clinical and Functional Assessment:**		
Symptoms and Signs	Asymptomatic, Palpitations, Chest Pain, dispnoea, fatigue, diziness, embolism complications or exacerbation of heart failure, weight loss, diarrhea.	Polytomous Qualitative
Conditions (comorbidity) and associated health disorders	High blood pressure, heart disease, mitral stenosis, mitral regurgitation, hypertrophic cardiomyopathy, pericarditis,congenital heart disease, previous cardiac surgery, lung diseases (pneumonia, lung cancer, pulmonary embolism, sarcoidosis), alcohol abuse, hyperthyroidism, bone and joint disorders, neurological, hearing, vision, feet, psychic, digestive, urinary, sleep disorders, other.	Polytomous Qualitative
	To classify chronic disorders we will use the electronic version of the *Second International Clasiffication of Primary Care* (ICPC-2-E) [31]	
Family History of AF	Parents, siblings, children	Polytomous Qualitative
Drug Consumption for Cardiovascular Disease	Name of the drug: The Anatomical Therapeutic Classification (AT) developed by the European Pharmaceutical Market Research Association (EPhMRA) [32]	Polytomous Qualitative
Number of Drugs	Drugs currently consumed	Discrete
		Quantitative
**Dependent:**		
**Peripheral Blood Pulse**	Radial pulse measuring according to the protocol: Regular/Irregular	Dichotomous Qualitative
**Atrial Fibrillation**	ECG according to the clinical protocol	Dichotomous Qualitative
Treatment	Pharmacological (Antiarrhythmic / Anticoagulation)/Non-pharmacological (Cardioversion)	Polytomous Qualitative

### -Statistical Analysis

Statistical analysis will be performed with the statistics analysis software EPIDAT 3.1 [29], SPSS [33], and WLwiN 2.02 [34]. It will include:

a. Descriptive Analysis: characteristics of the population under study (sociodemographic variables): central tendency, dispersion and position measures for quantitative variables; and tabulation and calculation of relative frequencies for qualitative variables. A calculation of major estimators with their corresponding 95% confidence intervals (CI95%) will be performed.

b. Bivariate Analysis: We will examine basal comparability between the two groups using the chi-square test or Fisher’s test (proportion comparison test), or by Student’s t-test (mean comparison test after testing normal distribution). Then, the AF dependent variable will be examined and crossed with the sociodemographic variables and other independent or prognostic variables of interest. On such purpose, we will use Pearson’s chi square tests or Fisher’s test (qualitative variables), and Student’s t-test or ANOVA (quantitative variables). All contrasts will be bilateral (p < 0.05).

c. Analysis of the relationship between the type of intervention and the primary variable of the study (AF): contingency tables, square chi test, relative risk estimation), attributable risk and number of subjects to be treated, with their respective CI95%.

d. Multivariate Analysis: A multi-level logistic regression or multiple logistic regression will be performed to compare effects of the strategy on both groups (Odds Ratio) adjusting the model for potential prognostic and / or confounding candidate variables. When multiple logistic regression is used, these variables will be introduced by the “Enter” technique. Significance will be > 0.10 according to Wald’s test.

### Protection against bias

The study is open, so the intervention cannot be masked. Therefore, both, patients and practitioners will know who is involved in the intervention, and this might condition their response (Hawthorne effect), especially in the control group. Just the fact that the participants are identified and express their wish to participate in the study, and since they are subject to more intensive monitoring than usual, might make them more willing to follow medical advice.

Information bias can occur through errors in FA identification on the ECG due the practitioner’s inadequate skills. Practitioners will previously take a training course. The degree of interobserver reliability and agreement on blood pulse measuring will be examined.

Another bias might occur in the selection of patients, as one of the groups might include more patients diagnosed with AF before the study period. Should this occur in the control group, the beneficial effect of screening might be overestimated. Randomization will help balance both groups.

## Discussión

### Impact of results

This study will assess the effectiveness of early detection of AF. If our hypothesis is confirmed, we expect Primary Care professionals to take a more proactive approach and adopt a new protocol when a patient meeting the established screening criteria is identified. Finally, we expect this measure to be incorporated into Clinical Practice Guidelines.

### Ethical considerations

The research project has been approved by the Ethics Committees of the Hospital de Mataró (Barcelona) and the Hospital Reina Sofía (Córdoba). Informed consent will be previously obtained from all participants. This project will be conducted under the provisions of the Declaration of Helsinki, the European Council agreement on Human Rights and Biomedicine, and the UNESCO Universal Declaration.

The design and development of this study will be performed within Best Practice and Confidentiality norms (protection of personal data) and other national and international laws in force regulating health research.

This trial is registered in ClinicalTrials.gob (NCT01291953).

### Organization and logistics of the study

Systematic tracking of the fieldwork will be performed by a technical instructor, a monthly phone conversation with every practitioner, and collecting recruitment and DCF data. Subsequently, a case-based reasoning will be submitted to each practitioner.

Coordination meetings with the project management team will be held via Second Life (http://secondlife.com/) at least once every two months over the study period.

## Competing interests

Non-financial competing interests.

## Authors’ contributions

MAMA and LAPT are the principal Investigators who conceived the study and led the study design and funding application. LAPT and JMBD contributed to the Statistical Analysis Plan. They coordinated the preparation of this manuscript. VGB, EMR, JRC, JAFG, ARV, JGL, RRM, CPT, AVM, JMPR, RMA, JAFG, MP, RMB, EBJ, MCS and LCC contributed to the study design, funding application, diffusion of the study, practitioner recruitment, study implementation and intervention development. They also contributed to writing the paper. MCL and IOC contributed to the study monitoring and to data and input collection. All authors contributed to, read and approved the final version of this manuscript.

## Pre-publication history

The pre-publication history for this paper can be accessed here:

http://www.biomedcentral.com/1471-2296/13/106/prepub

## References

[B1] Fibrilación auricular 20/10/2009-Guías Clínicas 2009 9 34 http://www.fisterra.com/guias2/fa.asp

[B2] Go AS Hylek EM Phillips KA Chang Y Henault LE Selby JV Singer DE Prevalence of diagnosed atrial fibrillation in adults: national implications for rhythm management and stroke prevention: the AnTicoagulation and Risk Factors in Atrial Fibrillation (ATRIA) Study JAMA 2001 285 2370 2375 10.1001/jama.285.18.2370 11343485

[B3] Furberg CD Psaty BM Manolio TA Gardin JM Smith VE Rautaharju PM Prevalence of atrial fibrillation in elderly subjects (the Cardiovascular Health Study) Am J Cardiol 1994 236 41 10.1016/0002-9149(94)90363-8 8037127

[B4] Psaty BM Manolio TA Kuller LH Kronmal RA Cushman M Fried LP Incidence of and risk factors for atrial fibrillation in older adults. Circulation» Circulation 1997 96 61 10.1161/01.CIR.96.1.61 9337224

[B5] Cea-Calvo L Redón J Lozano JV Fernández-Pérez C Martí-canales JC Llisterri JL González Esteban J Aznar J Prevalencia de fibrilación auricular en la población española de 60 o más años de edad Estudio PREV-ICTUS. Rev Esp Cardiol 2007 60 616 624 10.1157/13107118 17580050

[B6] Miyasaka Y Barnes ME Bailey KR Cha SS Gersh BJ Seward JB Tsang TS Mortality trends in patients diagnosed with first atrial fibrillation: a 21-year community-based study J Am Coll Cardiol 2007 49 986 992 10.1016/j.jacc.2006.10.062 17336723

[B7] Murphy NF Simpson CR Jhund PS Stewart S Kirkpatrick M Chalmers J MacIntyre K McMurray JJV A national survey of the prevalence, incidence, primary care burden and treatment of atrial fibrillation in Scotland Heart 2007 93 606 612 10.1136/hrt.2006.107573 17277353 PMC1955558

[B8] Heeringa J Van der Kuip DAM Hofman A Kors JA Van Herpen G Stricker BHC Prevalence, incidence and lifetime risk of atrial fibrillation: the Rotterdam study Eur Heart J 2006 27 949 953 16527828 10.1093/eurheartj/ehi825

[B9] Lloyd-Jones DM Wang TJ Leip EP Larson MG Levy D Vasan RS Lifetime Risk for Development of Atrial Fibrillation: the Framingham heart study Circulation 2004 110 1042 1046 10.1161/01.CIR.0000140263.20897.42 15313941

[B10] Wolf PA Dawber TR Thomas HE Jr Kannel WB Epidemiologic assessment of chronic atrial fibrillation and risk of stroke: the Framingham study Neurology 1978 28 973 977 10.1212/WNL.28.10.973 570666

[B11] Stewart S Hart CL Hole DJ McMurray JJ A population-based study of the long-term risks associated with atrial fibrillation: 20-year follow-up of the Renfrew/Paisley study Am J Med 2002 113 359 364 10.1016/S0002-9343(02)01236-6 12401529

[B12] Benjamin EJ Wolf PA D'Agostino RB Silbershatz H Kannel WB Levy D Impact of atrial fibrillation on the risk of death: the Framingham Heart Study Circulation 1998 98 946 952 10.1161/01.CIR.98.10.946 9737513

[B13] Wang TJ Larson MG Levy D Vasan RS Leip EP Wolf PA Temporal relations of atrial fibrillation and congestive heart failure and their joint influence on mortality: the Framingham Heart Study Circulation 2003 107 2920 2925 10.1161/01.CIR.0000072767.89944.6E 12771006

[B14] Hart RG Halperin JL Atrial fibrillation and thromboembolism: a decade of progress in stroke prevention Ann Intern Med 1999 131 688 695 10577332 10.7326/0003-4819-131-9-199911020-00010

[B15] Hamer ME Blumenthal JA McCarthy EA Phillips BG Pritchett EL Quality-of-life assessment in patients with paroxysmal atrial fibrillation or paroxysmal supraventricular tachycardia Am J Cardiol 1994 74 826 829 10.1016/0002-9149(94)90448-0 7942563

[B16] Friberg J Buch P Scharling H Gadsbphioll N Jensen GB Rising rates of hospital admissions for atrial fibrillation Epidemiology 2003 14 666 672 10.1097/01.ede.0000091649.26364.c0 14569181

[B17] Samuel Wann L Curtis AB Craig T Ellenbogen KA Lowe JE Mark NA ACCF/AHA/HRS Focused Update on the Management of Patients With Atrial Fibrillation (Updating the 2006 Guideline) A Report of the American College of Cardiology Foundation/American Heart Association Task Force on Practice Guidelines Circulation 2011 123 104 123 21173346 10.1161/CIR.0b013e3181fa3cf4

[B18] Los cardiólogos hacen un llamamiento para fomentar la concienciación y la educación sobre la fibrilación auricular Sociedad Española de Cardiologíahttp://www.secardiologia.es/actualidad/notas-de-prensa/2155-los-cardiologos-hacen-un-llamamiento-para-fomentar-la-concienciacion-y-la-educacion-sobre-la-fibrilacion-auricular

[B19] Mant J Fitzmaurice DA Hobbs FDR Jowett S Murray ET Holder R Accuracy of diagnosing atrial fibrillation on electrocardiogram by primary care practitioners and interpretative diagnostic software: analysis of data from screening for atrial fibrillation in the elderly (SAFE) trial BMJ 2007 335 380 10.1136/bmj.39227.551713.AE 17604299 PMC1952490

[B20] Sudlow M Rodgers H Kenny RA Thomson R Identification of patients with atrial fibrillation in general practice BMJ 1999 318 264 9915750 10.1136/bmj.318.7178.264 PMC1114740

[B21] Benjamin EJ Chen PS Bild DE Mascette AM Albert CM Alonso A Prevention of Atrial Fibrillation: Report From a National Heart, Lung, and Blood Institute Workshop Circulation 2009 119 606 618 10.1161/CIRCULATIONAHA.108.825380 19188521 PMC2635942

[B22] Koenig KL Screening for AF in Elders: Keep Your Finger on the Pulse J Watch Emergency Med 2007 August 30:3-3 [http://emergency-medicine.jwatch.org/cgi/content/full/2007/830/3]

[B23] Van Weert HCPM Diagnosing atrial fibrillation in general practice BMJ 2007 335 355 356 10.1136/bmj.39266.497396.BE 17717334 PMC1952519

[B24] Robledo E González P Burgos A García R Barasoain P Lorenzo A Estudio de prevalencia de la fibrilación auricular en la población de 65 años o más. Validez de la toma de pulso radial como cribado de fibrilación auricular SEMERGEN 2005 31 303 306

[B25] Fitzmaurice DA Hobbs FD Jowett S Mant J Murray ET Holder R Screening versus routine practice in detection of atrial fibrillation in patients aged 65 or over: cluster randomised controlled trial BMJ 2007 335 383 10.1136/bmj.39280.660567.55 17673732 PMC1952508

[B26] Wilson JMC Jungner G The principles and practices of screening for diseases. Public Health papers34 1968 Geneva, Switzerland: WHO

[B27] Hobbs FDR Fitzmaurice DA Mant J Murray E Jowett S Bryan S A randomised controlled trial and cost-effectiveness study of systematic screening (targeted and total population screening) versus routine practice for the detection of atrial fibrillation in people aged 65 and over: the SAFE study Health Technol Assess 2005 9 40 10.3310/hta9400 16202350

[B28] Guías de práctica clínica para el manejo de la fibrilación auricular Rev Esp Cardiol 2010 63 1483 e1 e83 http://www.revespcardiol.org/es/revistas/revista-espa%C3%B1ola-cardiologia-25/guias-practica-clinica-manejo-fibrilacion-auricular---13188310-guias-practica-clinica-2010

[B29] Programa EPIDAT Dirección Xeral de Innovación e Xestión da Saúde Pública (Edificio administrativo San Lázaro, s/n. 15703, Santiago de Compostela, Spain) and PAHO-WHO (525 Twenty-third St, NW, Washington, DC 20037)

[B30] Viana C Fibrilación auricular (fecha de la última revisión: 08/03/2012) 2012 http://www.fisterra.com/guias2/fa.asp

[B31] Second International Clasiffication of Primary Care (ICPC-2-E) http://www.who.int/classifications/icd/adaptations/icpc2/en/index.html

[B32] The Anatomical Therapeutic Classification (AT). European Pharmaceutical Market Research Association (EPhMRA) http://www.ephmra.org/classification/anatomical-classification.aspx

[B33] Programa estadístico SPSS Inc 11th Fl, Chicago, IL 60606L: 233 S Wacker Dr

[B34] Programa estadístico MLwiN Centre for Multilevel Modelling Senate House, Tyndall Ave, Bristol BS8 1TH, UK: University of Bristol

